# Targeting the PI3K/Akt/mTOR signalling pathway in Cystic Fibrosis

**DOI:** 10.1038/s41598-017-06588-z

**Published:** 2017-08-09

**Authors:** R. Reilly, M. S. Mroz, E. Dempsey, K. Wynne, S. J. Keely, E. F. McKone, C. Hiebel, C. Behl, J. A. Coppinger

**Affiliations:** 1 Conway Institute, University College Dublin, Belfield, Dublin 4 Ireland; 2 Royal College of Surgeons in Ireland, Beaumont Hospital, Dublin, 9 Ireland; 3 Royal College of Surgeons in Ireland, 123 St. Stephens’s Green, Dublin, 2 Ireland; 4 St Vincent’s University Hospital, Dublin, 4 Ireland; 5 Institute for Pathobiochemistry, University Medical Center of the Johannes Gutenberg- University Mainz, Frankfurt, Germany; 6 School of Biology and Environmental Science, University College Dublin, Belfield, Dublin 4 Ireland

## Abstract

Deletion of phenylalanine 508 of the cystic fibrosis transmembrane conductance regulator (ΔF508 CFTR) is a major cause of cystic fibrosis (CF), one of the most common inherited childhood diseases. ΔF508 CFTR is a trafficking mutant that is retained in the endoplasmic reticulum (ER) and unable to reach the plasma membrane. Efforts to enhance exit of ΔF508 CFTR from the ER and improve its trafficking are of utmost importance for the development of treatment strategies. Using protein interaction profiling and global bioinformatics analysis we revealed mammalian target of rapamycin (mTOR) signalling components to be associated with ∆F508 CFTR. Our results demonstrated upregulated mTOR activity in ΔF508 CF bronchial epithelial (CFBE41o-) cells. Inhibition of the Phosphatidylinositol 3-kinase/Akt/Mammalian Target of Rapamycin (PI3K/Akt/mTOR) pathway with 6 different inhibitors demonstrated an increase in CFTR stability and expression. Mechanistically, we discovered the most effective inhibitor, MK-2206 exerted a rescue effect by restoring autophagy in ΔF508 CFBE41o- cells. We identified Bcl-2-associated athanogene 3 (BAG3), a regulator of autophagy and aggresome clearance to be a potential mechanistic target of MK-2206. These data further link the CFTR defect to autophagy deficiency and demonstrate the potential of the PI3K/Akt/mTOR pathway for therapeutic targeting in CF.

## Introduction

CF is caused by mutations in the CFTR gene. CFTR is a chloride channel primarily responsible for facilitating conductance of chloride and other ions across epithelial membranes. The loss of a functional CFTR channel disrupts ionic homeostasis resulting in tenacious mucus production and a descent into a vicious cycle of chronic infection inflammation, and progressive lung fibrosis^1^. There are almost 2,000 different variants in the CFTR gene^2^ and up to 70% of CF patients contain at least one allele with a mutation at position 508 (c.1521_1523delCTT; commonly known as ΔF508), which results in the loss of Phe508 and disruption of the folding pathway of CFTR in the ER^3^. CFTR is synthesised in the ER and transport of CFTR involves chaperones that facilitate folding and trafficking from the ER to the cell surface membrane.

As ΔF508 CFTR fails to achieve a wild type fold, it does not engage with COPII ER export and is processed through ER-associated degradation (ERAD)^4, 5^. The inability of protein p.Phe508del (ΔF508 CFTR) to achieve a correct folded state disrupts cellular proteostasis networks which protect the cell from acute stress^6^. In situations of cellular stress where the 26S proteasome is compromised or overwhelmed, ubiquitinated ΔF508 CFTR is transported to a perinuclear location to form aggresomes^7^. Autophagy is important in clearing protein aggregates after overload of polyubiquitinated proteins. The accumulation of misfolded protein aggregates has been described in several human disorders and the PI3K/Akt/mTOR axis plays a central role in maintaining cellular proteostasis, with mTOR activation regulating autophagy^8^. ΔF508 CFTR is described as an aggresome prone protein and aggresome formation has been linked to defective autophagy in CF^9^.

In order to rationally develop therapeutic strategies to stimulate CFTR trafficking from the ER to the surface and maintain cellular proteostasis, it is critical to understand the protein interactions regulating CFTR transport. State of the art approaches such as mass spectrometry-based proteomics have been successful in identifying novel effectors of ΔF508 CFTR rescue from ERAD^10^. Silencing of key proteostatic chaperones led to a partial rescue of ΔF508 CFTR cell surface channel activity^10, 11^. Proteostasis regulators, such as Cystamine, have also been shown to rescue and stabilize functional ΔF508 CFTR protein, restoring autophagy *in vivo* ^12^. Clinically, progress has been made in recent years identifying therapeutics that target CFTR dysfunction in patients with specific mutations. However, small molecules that directly target the most common misfolded CFTR mutant, ΔF508, and improve its intracellular trafficking *in vitro*, have shown modest effects^13^. This study aimed to identify new therapeutic targets that will help address the unmet clinical need for CF patients with the ΔF508 mutation.

In this study, we describe a role for mTORC1/2 regulation in ΔF508 expressing CF cells. We observed increased mTOR activity in ΔF508 CF bronchial epithelial cells and discovered that inhibition of the PI3K/Akt/mTOR pathway improved CFTR stability and expression. We also showed that selected inhibitors, exerted rescue effects by restoring autophagy in ΔF508 expressing cells.

## Methods

### General Materials

Cystic Fibrosis Bronchial Epithelial cells (CFBE41o-) expressing ∆F508 CFTR mutation, HBE41o- cells possessing a functional WT CFTR allele, and isogenic CFTR null cells (CFBE41o-, null) were obtained from Prof’s. E Sorscher/J Clancy (University of Alabama, Birmingham). All other materials crucial to experimentation are included in Supplementary Information.

### Cell culture

WT HBE41o- and CFBE41o- (ΔF508 homozygous) cells were cultured in MEM (Minimum Essential Medium) supplemented with 10% FCS (Fetal Calf Serum), 2 mM glutamine and 1% penicillin/streptomycin. Cells were grown at 37 °C in 5% CO_2_. PI3K/Akt/mTOR inhibitors or DMSO (vehicle control) were incubated with cells for 48 hours at indicated concentrations. DMSO concentration never exceeded 0.1%. For Ussing chamber experiments, cells were plated onto Costar snapwell inserts (12 mm), as previously described^14^. Transepithelial resistance (R_T_) was monitored and when the cells achieved 500–1,000 Ω cm^2^, they were used for experimentation. Adequate R_T_ was usually achieved after 7 days in culture. For autophagy experiments, ΔF508 CFBE41o- cells were incubated in serum depleted media for 24 hours and treated with 10 µM MG132 to detect aggresomes.

### siRNA Transfections

ΔF508 CFBE41o- cells were transfected with 5 nM siRNA using 4 µl Lipofectamine (Invitrogen, Grand Island, NY) as described previously^15^. Briefly, lipid-siRNA complexes were prepared in serum free media and added to cell suspensions in culture medium with 10% FCS. After 24 h, the medium was replaced. The target sequence was Bag3: 5′-GCCUGAAAACAAACCAGAATT-3′.

### Co-interacting Protein Identification Technology (Co-PIT) and LC-MS/MS sample preparation

This protocol was carried out using a method previously described with minor modifications^16^. Anti-mouse CFTR 596 antibody was coupled to Dynabeads^TM^ protein G (Thermo Scientific, Rockford, IL) and crosslinked with 25 mM dimethyl pimelimidate. In parallel, WT HBE41o- and ΔF508 CFBE41o- cells were grown to confluence, washed in PBS and lysed on ice using 1% NP-40 solution containing protease inhibitor cocktail, pH 7.4, and phosphatase inhibitor cocktail. After sonication and clarification at 1,000 rpm for 10 mins, the pre-cleared cell extracts (4 mg of protein for each condition) were incubated with crosslinked antibody-dynabead complex overnight at 4 °C. Proteins bound to the antibody were recovered by washing three times with immunoprecipitation (IP) lysis buffer and eluted by incubation at 37 °C in 8 M urea with agitation on two occasions. Endogenous immunoprecipitates were isolated by methanol-chloroform precipitation. The eluate was resuspended in 100 mM TRIS pH 8.5.

0.2% Rapigest (Waters, Milford, MA) was added to the eluted proteins and reduced with 5 mM dithiothreitol. The samples were digested overnight at 37 °C with 2 µg Sequencing Grade Modified Trypsin (Promega, Madison, WI). Proteolysis was acidified with 5% formic acid. The peptides were isolated using a stop and go extraction protocol (STAGE)^17^. Briefly, the samples were washed in buffer A (0.1% TFA), immobilized on a C18 column, washed in buffer B (50% ACN, 0.1% TFA) and eluted in buffer C (1% TFA). Non-specific proteins were accounted for by performing immunoprecipitations IPs with CFTR null CFBE41o- cells.

### Liquid Chromatography Mass Spectrometry Analysis

#### Chromatography

Tryptic digested peptide mixtures from the Endogenous IP samples were run on a Thermo Scientific Q Exactive mass spectrometer connected to a Dionex Ultimate 3000 (RSLC nano) chromatography system. Tryptic peptides were re-suspended in 0.1% formic acid. Each sample was loaded onto Biobasic Picotip Emitter (120 mm length, 75 μm ID) packed with Reprocil Pur C18 (1.9 μm) reverse phase media and was separated by an increasing acetonitrile gradient over 59.5 min at a flow rate of 250 nL/min. From 0–15 minutes the sample is loaded on to the column at 500 nl/min in 100% Buffer A (97% water, 2.5% acetonitrile, 0.5% acetic acid). From 15–16 minutes Buffer B (97% acetonitrile, 2.5% water, 0.5% acetic acid) increased from 1–2% and the flow rate decreased from 500–250 nl/min, from 16–64 minutes Buffer B increased from 2–35% at a flow rate of 250 nl/min, from 64–66 minutes Buffer B increased from 35–90% and the flow rate increased from 250–600 nl/min, from 66–70 minutes Buffer B remained at 90% and a flow rate of 600 nl/min, from 70–71 minutes Buffer B decreased from 90–1% at a flow rate of 600 nl/min, from 71–71.5 minutes Buffer B remained at 1%, at a flow rate of 600 nl/min.

#### Ionisation and Mass Spectrometry

The mass spectrometer was operated in positive ion mode with a capillary temperature of 220 °C, and with a potential of 2.1 kV applied to the frit. The heated capillary was kept at 150 °C. The capillary voltage was set at 45 V, and the tube lens was offset at 100 V. The mass spectrometer was operated in data-dependent positive ion-mode A top 12 method was used. Full MS scans were acquired in the Orbitrap mass analyzer over the range m/z 300–1600 with a mass resolution of 70000 (at m/z 200). The target value was 3.00 E+06. The twelve most intense peaks were fragmented in the high energy collision dissociation cell with a normalized collision energy of 27%, and tandem mass spectra were acquired in the Orbitrap mass analyzer with a mass resolution of 17500 at m/z 200.

#### Analysis of Tandem Mass Spectra

The Q-Exactive MS raw data files were *de novo* sequenced and searched against a Human UniProtKB database Release 2013_07, 20,266 entries using the search engine PEAKS Studio 6, for peptides cleaved with trypsin. Each peptide used for protein identification met specific Peaks parameters, for example; only peptide scores that corresponded to a false discovery rate (FDR) of ≤1% were accepted from the Peaks database search. The database searching parameters included up to two missed cleavages allowed for full tryptic digestion, and a precursor ion mass tolerance of 20 ppm. A fragment ion error tolerance of 0.03 Da was also included in the search parameters. A fixed modification of cysteine (57.02146) and up to 669 variable modifications were included in the Peaks PTM search.

### Ingenuity and GO Analysis

Proteins identified by mass spectrometry were displayed in Venn Diagrams using Venny Software (http://bioinfogp.cnb.csic.es/tools/venny). Proteins identified by mass spectrometry were overlaid onto a global molecular network developed from information contained in the ingenuity knowledge base (Ingenuity Systems®, http://www.ingenuity.com, content version 12402621, release date: 2012-03-09). The top ten canonical pathways significantly (p < 0.05) associated with WT and ΔF508 CFTR are listed. Molecular Functions were also described for the identified proteins using information contained within the Gene Ontology Database (http://geneontology.org).

### Protein extraction and immunoblotting

For immunoprecipitation, cell extracts were prepared in IP lysis buffer (1% sodium dodecyl sulfate (SDS), mammalian protease inhibitor cocktail, pH 7.4, and phosphatase inhibitor cocktail). Protein concentration was determined by the BCA assay (Thermo Scientific, Rockford, IL). For Western blotting, equal amounts of protein were separated by SDS-polyacrylamide gel electrophoresis and transferred onto polyvinylidene fluoride membranes. Membranes were blocked (0.05% Tween 20 and 5% non-fat dry milk or 3% BSA) prior to incubation with antibodies. Horseradish-peroxidase-conjugated secondary antibodies were visualized using SuperSignal West Pico or Femto reagents (Thermo Scientific, Rockford, IL).

### Immunofluorescence

Cells growing on glass coverslips in 6-well plates were fixed with 4% formaldehyde for 15 minutes. Cells were incubated with PBS (phosphate buffered saline, 0.1 M, pH 7.4) containing 4.5% BSA and 0.2% Triton X-100, for 2 h at room temperature. This was followed by overnight incubation at 4 °C with primary antibodies targeting CFTR and LC3 (at 1:200 and 1:170 dilutions, respectively). Secondary antibody conjugated to Alexa Fluor 594 and Alexa Fluor 488 were used to detect bound primary antibodies (Molecular Probes, Grand Island, NY). Prolong Gold anti-fade reagent (Life Technologies, Carlsbad, CA) was applied directly to fluorescently-labelled cells before mounting coverslips. Stained cells were imaged by confocal microscopy with an LSM 510 microscope (Carl Zeiss GmbH, Oberkochen, Germany) using the same settings for each preparation. Aggresomes were detected by microscopy using the Aggresome Detection Reagent Dye (Enzo LifeSciences, Exeter, UK) as per manufacturer’s instructions

### Measurements of short-circuit current (Isc)

Monolayers of ΔF508 CFBE41o- cells, grown on Snapwell supports, were mounted in Ussing chambers (p2300; Physiologic Instruments, San Diego, CA). The cells were bathed bilaterally with Ringer-bicarbonate solution, containing 120 mM NaCl, 25 mM NaHCO_3_, 3.3 mM KH_2_PO_4_, 0.8 mM K_2_HPO_4_, 1.2 mM MgCl_2_, 1.2 mM CaCl_2_, and 10 mM glucose. A chloride ion (Cl^−^) gradient was created by substituting NaCl for equimolar D-gluconic acid sodium salt in the apical bathing solution^14, 18^. The inserts were continuously gassed with 5% CO_2_-95% O_2_ and the temperature maintained at 37 °C^19^. Following set up, cells were equilibrated for 15 mins prior to experimentation. Cells were voltage-clamped to zero potential difference by the application of a short circuit current (Isc, measured in µA/cm^2^) using an EVC4000 voltage clamp (World Precision Instruments, Sarasota, FL). The maximal changes in Isc (∆Isc) after treatment with forskolin (10 µM), genistein (50 µM) or VX-770 (10 µM), were recorded.

### Statistical analysis

All quantified data are presented as the mean ± SD for at least three independent experiments. For each experiment, the statistical tests are indicated in the results section. Student paired *t-test* and unpaired *t-test* analysis was conducted using Prism 5 (Graphpad Software, La Jolla, CA). Unpaired *t-tests* were used for analysing 2 different variables (WT, ΔF508). Paired *t-test* analysis were used to compare different timepoints of the same variable (before and after drug). Quantification of protein expression for all blots was performed using Image J densitometry software (https://imagej.nih.gov/ij) with protein expression values normalised to the loading control (GAPDH).

## Results

### Protein interaction profiling reveals a ΔF508 CFTR specific interactome

**Figure 1:**
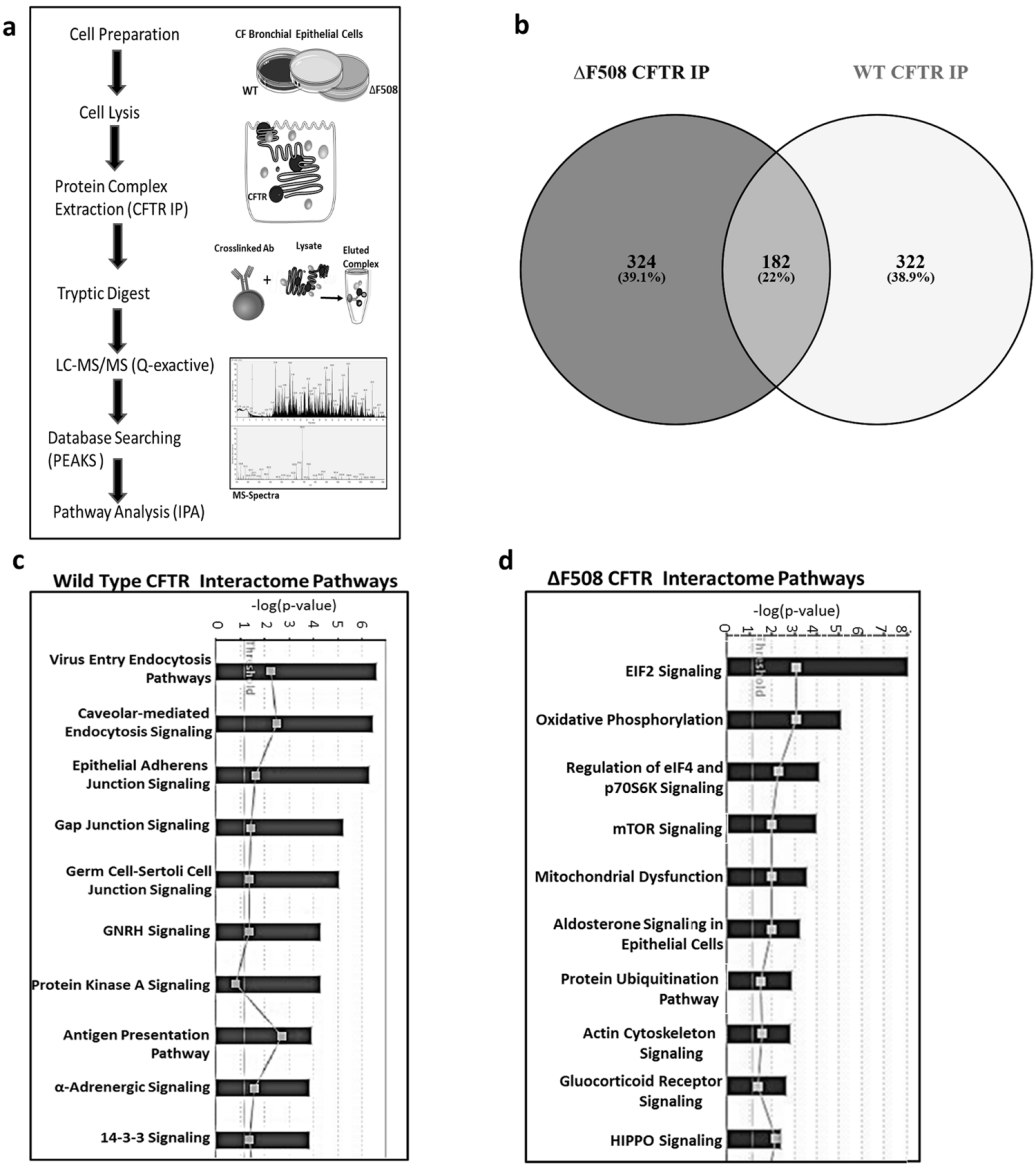
CFTR interactome in HBE41o- and CFBE41o- cells. (**a**) CFTR was isolated from WT HBE41o- and ΔF508 CFBE41o- cells and the resulting CFTR immunocomplexes were subjected to mass spectrometry analysis. An experimental schematic is shown. An experimental schematic is shown. The schematic was produced, in part, by using Servier Medical Art, (www.servier.com/Powerpoint-image-bank). (**b**) The number of proteins identified by mass spectrometry were displayed using Venny Software. Proteins identified in the CFTR interactomes were overlaid onto global molecular networks, developed from information contained in the Ingenuity knowledge database. Canonical pathways significantly associated with proteins (p < 0.05) in the WT CFTR interactome (**c)** and ΔF508 CFTR interactome (**d**) are displayed.

To define global protein interactions involved in CFTR trafficking and function in exocytic and endocytic pathways, CFTR-containing protein complexes were immunoprecipiated from CFBE41o- cells and HBE41o- cells expressing ΔF508 CFTR and wild-type CFTR respectively and protease digested (Fig. 1a). The composition of the peptide mixture was determined using mass spectrometry. Proteins identified in the CFTR immunoprecipitates that were not observed in ΔF508 CFBE41o- cells expressing null CFTR were identified as real interactors. Stringent criteria were used to identify CFTR interacting proteins, at least two peptides per protein were required and only peptide scores that corresponded to a false discovery rate of ≤1% were accepted using PEAKS software. This was performed in triplicate. Proteins that were identified in at least two biological replicates were considered for further analysis. Using these criteria, a total of 506 proteins were identified in the ΔF508 CFTR immunoprecipitates and 504 in WT (Fig. 1b). GO analysis was performed and protein class and molecular functions are listed (Supplementary Table S1). In order to analyse which signalling pathways were associated with CFTR, proteins were overlaid onto global molecular networks, developed from information contained in the Ingenuity knowledge database. Significant associations with known canonical signalling pathways were determined (p < 0.05). Ingenuity Pathway Analysis analysis revealed that different pathways were represented significantly in the ΔF508 and WT CFTR interactomes. In the CFTR WT interactome there were more proteins associated with endocytic signalling, PKA signalling and antigen presentation (Fig. 1c). In the ΔF508 CFTR interactome an increase in proteins involved in ubiquitination and mitochondrial dysfunction was observed. Interestingly, we observed an association between eukaryotic intitation factor (EIF)2 and mTOR signalling with ΔF508 CFTR (Fig. 1d). While components of these pathways were identified in both CFTR interactomes, there was a more significant representation in the ΔF508 CFTR interactome (Supplementary Table S1).

### CFTR associates with the mTORC1/2 complex

**Figure 2:**
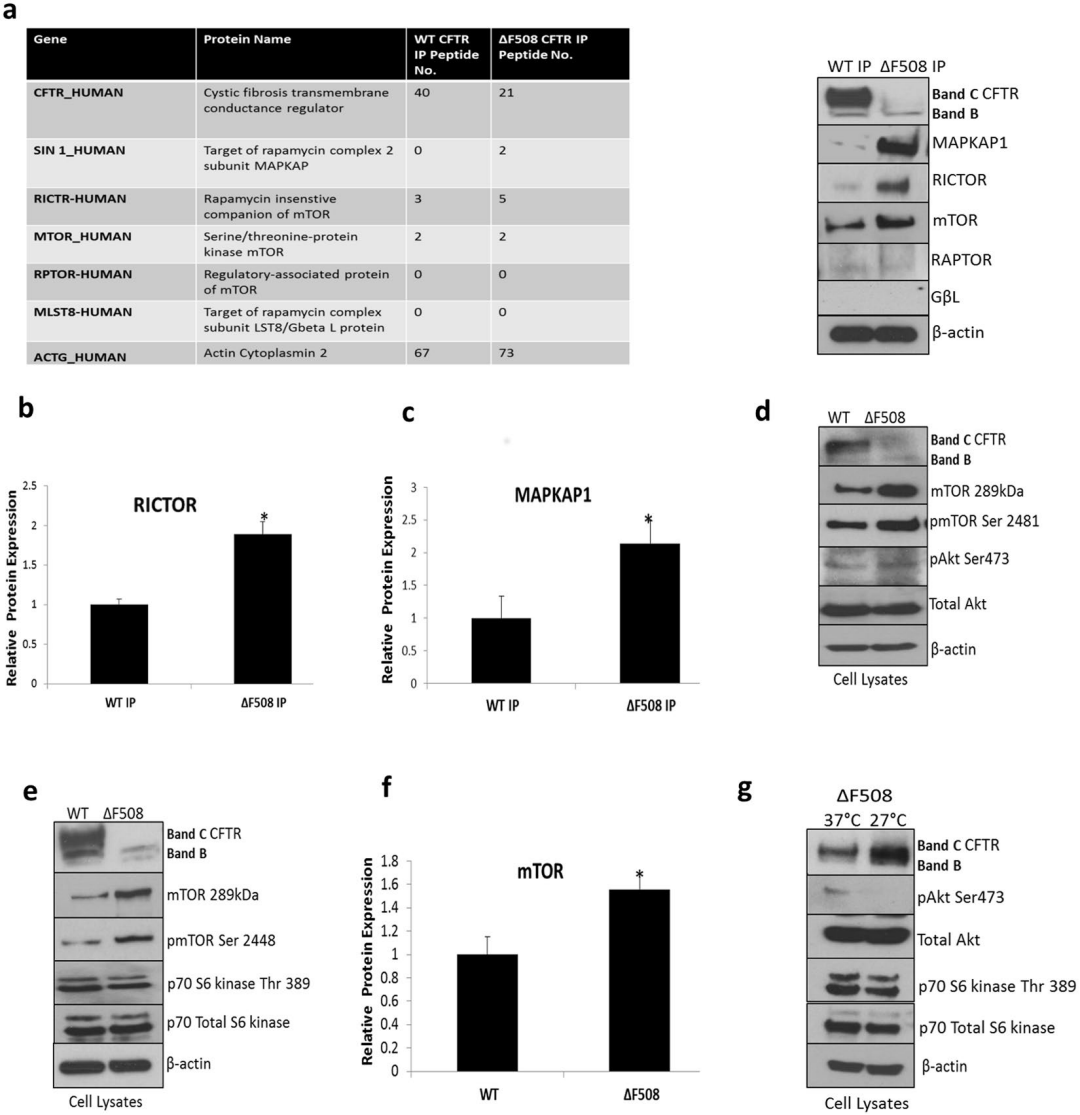
PI3K/AKT/mTOR signalling molecules associate with ΔF508 CFTR interactome. (**a**) CFTR was immunoprecipitated from WT HBE41o- and ΔF508 CFBE41o- cells and immunoblotting was performed for mTORC1/2 components, RICTOR, RAPTOR, mTOR, MAPKAP1 and GβL as well as CFTR and β-actin to confirm mass spectrometry observations (table left). A quantitative graph of (**b**) RICTOR and (**c**) MAPKAP1 in WT and ΔF508 CFTR immunoprecipitates. The results shown are representative of three independent experiments; the histograms represent the average and the error bars represent the standard deviation of the means. Statistical significance was examined using unpaired T-test analysis. Asterisk *represents p < 0.05. (**d)** Protein expression of mTOR, pmTOR Ser 2481, pAkt Ser 473 and total Akt (mTORC2) were determined by immunoblotting in WT HBE41o- and ΔF508 CFBE41o- cell lysates in addition to CFTR and β-actin. (**e**) Protein expression of mTOR, pmTOR Ser 2448, p70 S6 kinase Thr 389 and total 70 S6 kinase (mTORC1) were determined by immunoblotting in WT HBE41o- and ΔF508 CFBE41o- cell lysates in addition to CFTR and β-actin. (**f**) A quantitative graph of mTOR from WT HBE41o- and ΔF508 CFBE41o- cell lysates, the results shown are representative of three independent experiments; the histograms represent the average and the error bars represent the standard deviation of the means. Statistical significance was tested by unpaired T-test analysis. Asterisk* represents p < 0.05 (**g**). Protein expression of pAkt Ser 473, p70 S6 kinase Thr 389, Akt and p70 S6 kinase were determined by immunoblotting in ΔF508 CFBE41o- (37 °C) and ΔF508 CFBE41o- cells temperature shifted to 27 °C in addition to CFTR and β-actin.

Several components of the mTORC1/2 complex and downstream EIF signalling complex were identified to interact with CFTR using mass spectrometry, including mTOR, MAPKAP1, EIF3F, EIF4G2, EIF4A3, EIF4A1, RICTOR, EIF4G1 (Supplementary Table S1). In order to validate these findings, we performed immunoblot analysis on core members of the mTORC1/2 complexes identified in our protein interaction profiling screen, including 1. mTOR, 2. Rapamycin-insensitive companion of mTOR (RICTOR), 3. Regulatory-associated protein of mTOR (RAPTOR), 4. Target of rapamycin complex 2 subunit MAPKAP1 (MAPKAP1) and 5. GβL- target of rapamycin complex LST8 in ΔF508 and WT CFTR immunocomplexes. We visually observed an association between mTOR and both ΔF508 and WT CFTR, and a more pronounced association between RICTOR and MAPKAP1 and ΔF508 CFTR. RAPTOR and GβL were not associated with CFTR (Fig. 2a) in this study. RICTOR, MAPKAP1 and mTOR expression were quantified in triplicate. An increase in RICTOR expression (1.89 ± 0.34) in the ΔF508 CFTR IP was observed relative to WT (1.0 ± 0.14) (Fig. 2b). A significant increase in MAPKAP1 expression in the ΔF508 CFTR IP relative to WT was also observed (Fig. 2c) (p < 0.05). The mTOR protein was not significantly upregulated relative to WT. In order to determine if the interaction was direct or indirect, we performed a reverse IP for RICTOR in ΔF508 CFBE41o- and WT HBE41o- cells. We did not find CFTR present in the RICTOR IP but identified several chaperones, including Hsp70, which has been reported to bind both RICTOR and CFTR (Supplementary Fig. S1). In order to determine if mTORC2 is activated in ΔF508 CFBE41o- cell lysates, we measured expression of mTOR and phosphorylation of the mTOR protein at serine 2481. Additionally, we measured phosphorylation of Akt at Ser473. Activation of mTORC2 was present in ΔF508 CFBE41o- cell lysates (Fig. 2d). Activation of mTORC1 was also confirmed by measuring phosphorylation at Ser 2448 and p70S6 kinase (Fig. 2e). mTOR expression was quantified and a significant (p < 0.05) increase in mTOR protein expression (1.57 ± 0.1) was observed in ΔF508 CFBE41o- cell lysates relative to WT HBE41o- cells (1.0 ± 0.10) (Fig. 2f). Downstream activation of mTORC1/2 was also measured under temperature shift conditions and a decrease in phosphorylation of Akt Ser 473 (mTORC2) and p70 S6 kinase (mTORC1) was observed under these conditions (Fig. 2g).

### Targeting the PI3K/Akt/mTOR complex improves CFTR stability and function

**Figure 3:**
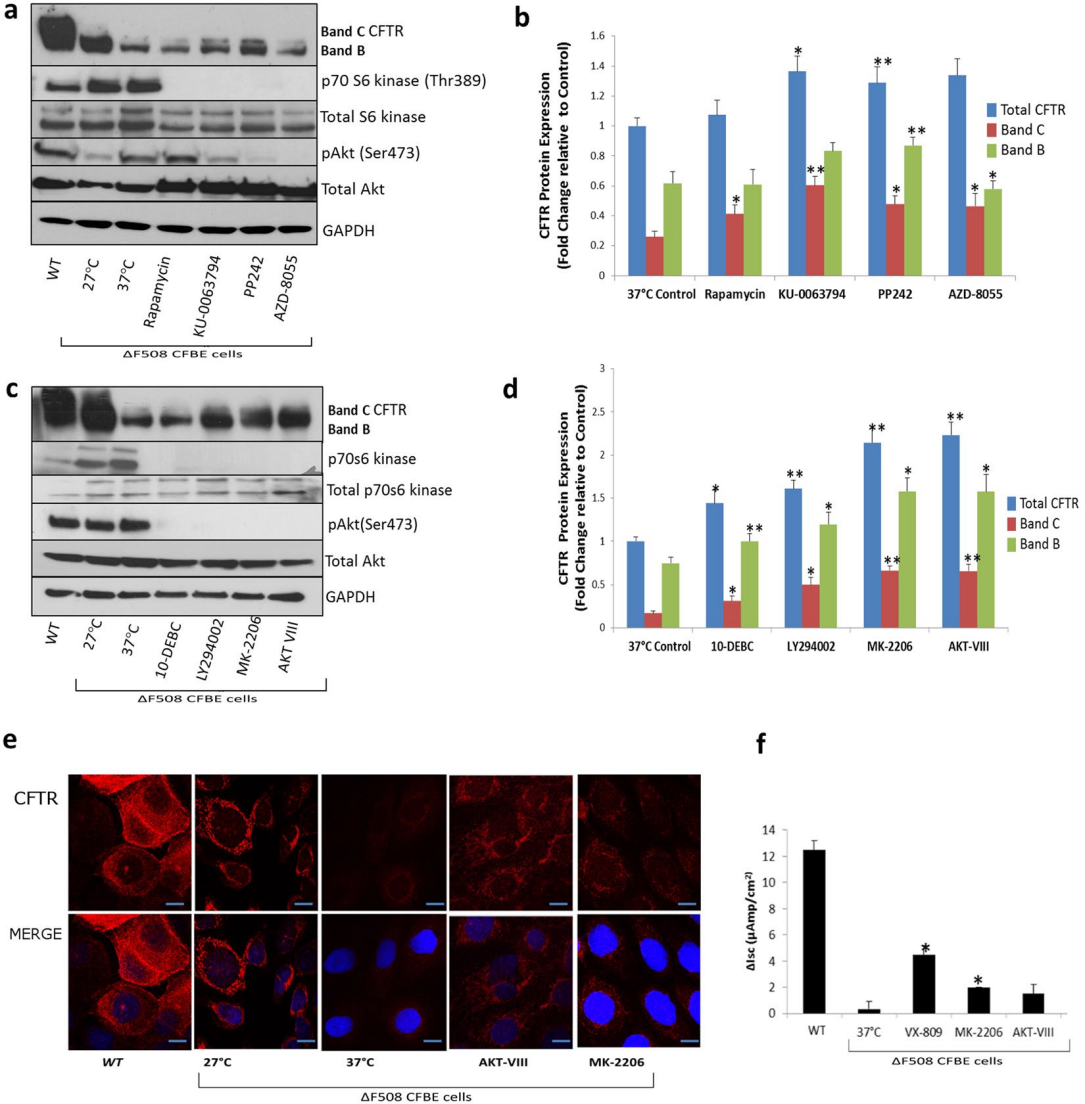
Targeting the PI3K/Akt/mTOR complex improves CFTR stability and function. (**a)** ΔF508 CFBE41o- cells (37 °C) were treated with Rapamycin, AZD-8055, PP-242, KU-0063794 (Set 1 inhibitors) and immunoblotting was performed for CFTR, pAkt Ser 473, p70 S6 kinase Thr389, Akt and p70 S6 kinase and GAPDH. WT HBE41o- cells and ΔF508 CFBE41o- cells incubated at 27 °C were included as positive controls and ΔF508 CFBE41o- cells incubated at 37 °C were included as a negative control. (**b**) A quantitative graph of CFTR (Total, Band B, Band C) from CFBE41o- cell lysates treated with Set 1 inhibitors. The results shown are representative of three independent experiments; the histograms represent the average and the error bars represent the standard deviation of the means. Statistical significance was examined using a two-tailed paired T-test analysis. Asterisk* represents p < 0.05, **represents p < 0.01. (**c)** ΔF508 CFBE41o cells (37 °C) were treated with LY-294002, 10-DEBC, MK-2206 and AKT-VIII and immunoblotting was performed for CFTR, pAKT Ser473, p70 S6 kinase Thr389, Akt and p70 S6 kinase and GAPDH. WT HBE41o- cells and ΔF508 CFBE41o- cells incubated at 27 °C were included as positive controls and ΔF508 CFBE41o- incubated at 37 °C were used as a negative control. (**d)** A quantitative graph of CFTR (Total, Band B, Band C) from ΔF508 CFBE41o lysates treated with Set 2 inhibitors. The results shown are representative of three independent experiments; the histograms represent the average and the error bars represent the standard deviation of the means. Statistical significance was examined using a two-tailed paired T-test analysis. Asterisk* represents p < 0.05, **represents p < 0.01. (**e)** Fluorescent detection was used to visualise CFTR (red- 549 nm) in ΔF508 CFBE41o- cells treated with MK-2206, AKT-VIII along with a negative control ΔF508 37 °C, positive controls ΔF508 27 °C and WT HBE41o- cells. Nuclei were stained with DAPI (blue- 358 nm). Scale bar is 10 µm. (**f**) Bar graph representing the change in Isc (ΔI_sc_) after forskolin (10 µM) and genistein (50 µM) addition relative to baseline readings in CFBE41o- cells treated with MK-2206, AKT-VIII, a negative control ΔF508 37 °C, positive controls VX-809 treatment and WT HBE41o- cells. Statistical significance was tested by a two-tailed paired T-test analysis. Asterisk* represents p < 0.05.

Based on our findings, we addressed the hypothesis that inhibition of mTORC1 or mTORC2 complexes would improve CFTR stability or export. A selection of kinase inhibitors was used to evaluate their ability to restore CFTR to the surface in ΔF508 CFBE41o- cells. Inhibitors were selected on the basis of their molecular targets and initial concentrations employed were selected based on previous literature reports^20–27^. The first set of inhibitors targeted mTORC1 alone (rapamycin) or targeted both mTORC1 and 2 complexes (AZD-8055, PP-242, KU-0063794). CFTR expression was measured by immunoblotting in ΔF508 CFBE41o- cells after drug treatment (2.5 µM). WT HBE41o- cells and ΔF508 CFBE41o- cells under temperature shift control (27 °C) were included. Phosphorylation of serine 473 on Akt, a marker of mTORC2 activation, and phosphorylation of p70S6 kinase at threonine 389, as a downstream marker of mTORC1 activation, were measured to ensure the complexes were effectively inhibited (Fig. 3a). Immunoblotting was performed in triplicate and we quantified the levels of total CFTR, Band B, and Band C relative to GAPDH (Fig. 3b). A small, but significant increase in total CFTR (approx. 1.3-fold) was observed upon treatment with PP-242 (1.26 ± 0.1, p < 0.01), and KU-0063794 (1.34 ± 0.1, p < 0.05) relative to 37 °C control (1.0 ± 0.07). In order to test more drugs acting on this pathway, we examined a second set of inhibitors targeting upstream of mTORC1/2 complexes. These included LY-294002, a PI3 kinase inhibitor, 10-DEBC, MK-2066, and AKT-VIII, which target Akt. ΔF508 CFBE41o- cells were treated AKT-VIII and MK-2206 (2.5 µM) for 48 hours and with LY-294002 (20 µM) and 10-DEBC (1.5 µM) for 24 hours to maintain viability. Levels of CFTR were then quantified as above. A significant (p < 0.05) increase in total CFTR, Band B and Band C (1.5–2 fold) was observed upon treatment with all drugs, with MK-2206 (2.14 ± 0.16) and AKT-VIII (2.22 ± 0.15) having the strongest effects relative to 37 °C ΔF508 CFBE41o- cell control (1.0 ± 0.05), demonstrating that inhibition of Akt could improve ΔF508 CFTR stability and export. We performed concentration-response curves and observed an increase in CFTR stability at higher drug concentrations (Supplementary Fig. S2) with dual mTOR inhibitors, AZD-8055/KU-0063794 and Akt inhibitors, MK-2206/AKT-VIII. However, we proceeded with lower concentrations (2.5 µM) to ensure cell viability was not compromised.

In order to further validate changes in CFTR expression after drug treatments, we performed confocal microscopy on ΔF508 CFBE41o- cells treated with four of the inhibitors that improved CFTR expression (Fig. 3e). CFTR was localised predominately in the cytoplasm and at the cell surface. A small increase in CFTR expression was observed after treatment with KU-0063794 and AZD-8055 (Supplementary Fig. S3) relative to the ΔF508-37 °C control and a greater level of CFTR expression was recovered after MK-2206 and AKT-VIII treatment (Fig. 3e), indicating again that targeting the PI3K/Akt/mTOR complex improves CFTR stability. In order to determine if CFTR function improved after drug treatment, measurements of Isc across voltage clamped monolayers of WT HBE41o- and ΔF508 CFBE41o- cells were performed in Ussing chambers. A notable increase in FSK (10 µM)-stimulated channel activity (~3-4 fold) was observed upon treatment with MK-2206 or AKT-VIII. The increase in channel activity with MK-2206 was significant (p < 0.05). VX-809 (currently used in ΔF508 CFTR correction therapy) was included as a control (Fig. 3f).

### Inhibitors of the PI3K/Akt/mTOR pathway improve CFTR recovery by stimulating autophagy

**Figure 4:**
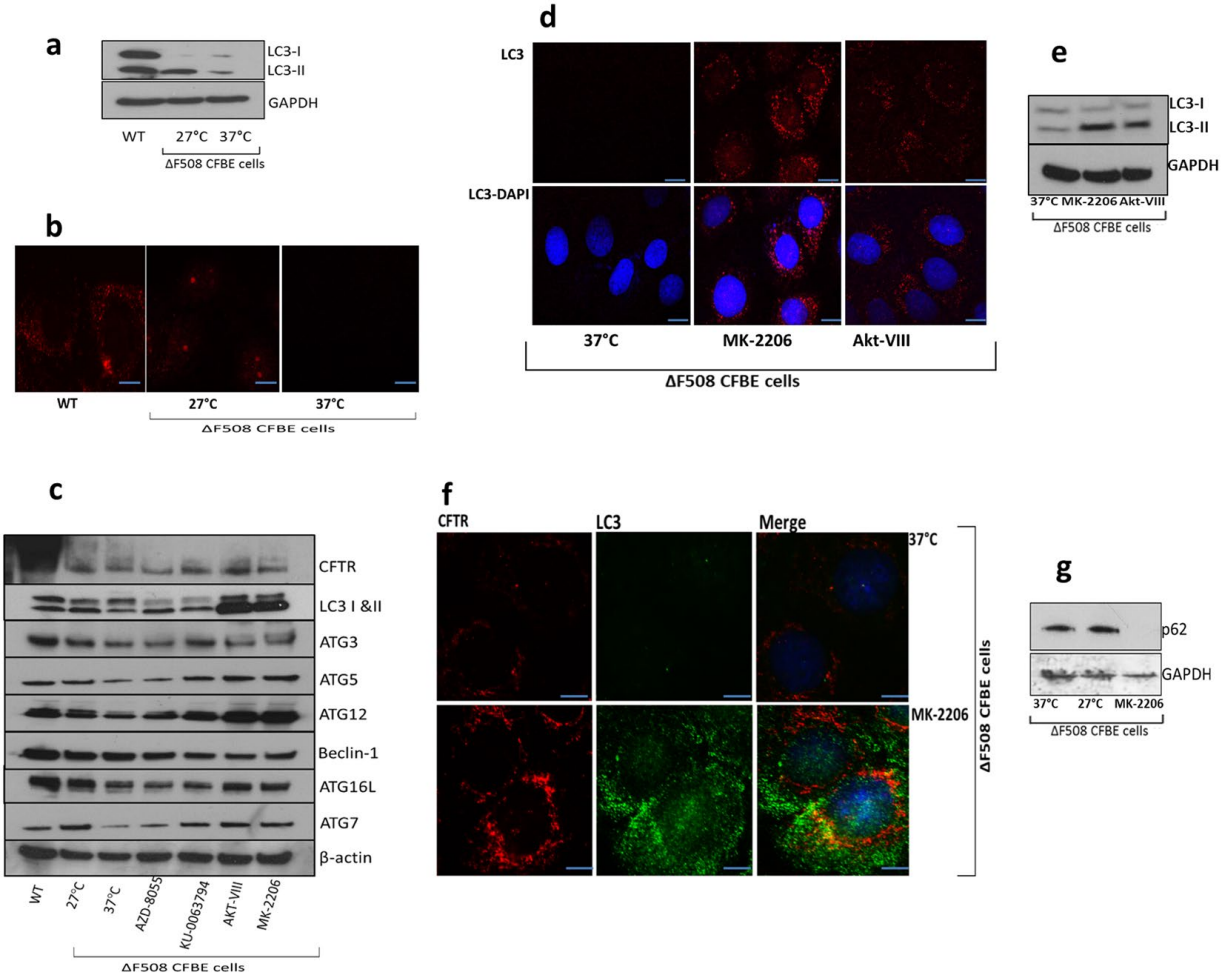
Autophagy is restored in ΔF508 CFBE41o- cells upon treatment with MK-2206 and AKT-VIII. (**a**) Protein expression of LC3-I, LC3-II was determined by immunoblotting in WT HBE41o- cells, ΔF508 CFBE41o- cells (37 °C) and ΔF508 CFBE41o- cells temperature shifted to 27 °C. (**b**) Subcellular localization of LC3 (red- 549 nm) was detected in WT HBE41o- cells, ΔF508 CFBE41o- cells (37 °C) and CFBE41o- cells temperature shifted to 27 °C. (**c)** ΔF508 CFBE41o- cells (37 °C) were treated with AZD-8055, KU-0063794, AKT-VIII and MK-2206 and protein expression of autophagy markers was determined by immunoblotting (LC3-I and -II, ATG3, ATG5, ATG7, ATG12, ATG16l and beclin-1) in addition to CFTR and β-actin. WT HBE41o- cells and ΔF508 CFBE41o- cells at 27 °C were included as positive controls and ΔF508 CFBE41o- cells at 37 °C were included as negative controls. (**d**) Fluorescent detection was used to visualise LC3 (red- 549 nm) in ΔF508 CFBE41o- (37 °C) treated with MK-2206 and AKT-VIII (**e**) ΔF508 CFBE41o- cells (37 °C) were treated with AKT-VIII and MK-2206 along with negative control ΔF508 CFBE41o- cells at 37 °C and protein expression of LC3-I, LC3-II and GAPDH was determined by immunoblotting. (**f**) Co-localisation of CFTR (red- 549 nm) and LC3 (green- 488 nm) in ΔF508 CFBE41o- cells treated with MK-2206 and a negative control CFBE41o- cells at 37 °C. Nuclei were stained with DAPI (blue- 358 nm). Scale bar is 10 µm. (**g)** Protein expression of p62 was determined in ΔF508 CFBE41o- cells (37 °C), ΔF508 CFB41o-E cells (27 °C) and ΔF508 CFBE41o- cells treated with MK-2206.

We serum starved human ΔF508 CFBE41o- cells alone and under temperature corrected conditions (27 °C) to stimulate autophagy. We measured the expression of LC3, a marker of autophagosome maturation under WT, control (37 °C) and temperature corrected rescue (27 °C) conditions by immunoblotting (Fig. 4a). There was increased expression of LC3B (II) detected in ΔF508 temperature corrected ΔF508 CFBE41o- cells and WT HBE41o- cells relative to ΔF508 control (Fig. 4a). To confirm this observation, we used immunofluorescence and observed minimal LC3 puncta in CFBE41o- cells and a higher number of LC3 puncta observed in the temperature corrected control and WT HBE41o- WT CFTR expressing cells (Fig. 4b) confirming autophagy is defective in ΔF508 CFBE41o- cells. Several inhibitors of the PI3K/Akt/mTOR pathway are reported to stimulate autophagy. Therefore, in order to determine if any of our target inhibitors could improve CFTR trafficking by stimulating autophagy, we measured a panel of autophagy markers in ΔF508 CFBE41o- cells after treatment with KU-0063794, AZD-8055, MK-2206, and AKT-VIII (Fig. 4c). A considerable increase in protein expression was observed for pre-autophagosome markers of vesicle expansion, the autophagy related genes (ATG5, ATG7, ATG12, ATG16l). These changes were most strongly observed in the presence of MK-2206 and AKT-VIII. Autophagosome formation was confirmed further by measuring LC3 expression. There was a marked increase in LC3 puncta in CFBE41o- cells treated with MK-2206 and AKT-VIII compared to the control (Fig. 4d). An increased presence of LC3B-II was also noted with MK-2206 and AKT-VIII treatment (Fig. 4e). In order to investigate if autophagy rescue improves CFTR expression in autophagosomes, we examined the co-localisation of CFTR and LC3 in ΔF508 CFBE41o- cells treated with MK-2206. Co-localisation was observed at the peripheral of the cytoplasm (yellow staining) (Fig. 4f) in ΔF508 CFBE41o- cells treated with MK-2206. Additionally, HBE41o- cells expressing WT CFTR and LC3 are included in Supplementary Fig. S4. We also observed a decrease in p62 expression in ΔF508 CFBE41o- cells treated with MK-2206 (Fig. 4g) and a decrease in CFTR ΔF508 aggregates upon MK-2206 treatment (Supplementary Fig. S5).

### MK-2206 modifies BAG3 expression which reduces CFTR ΔF508 aggregates

**Figure 5:**
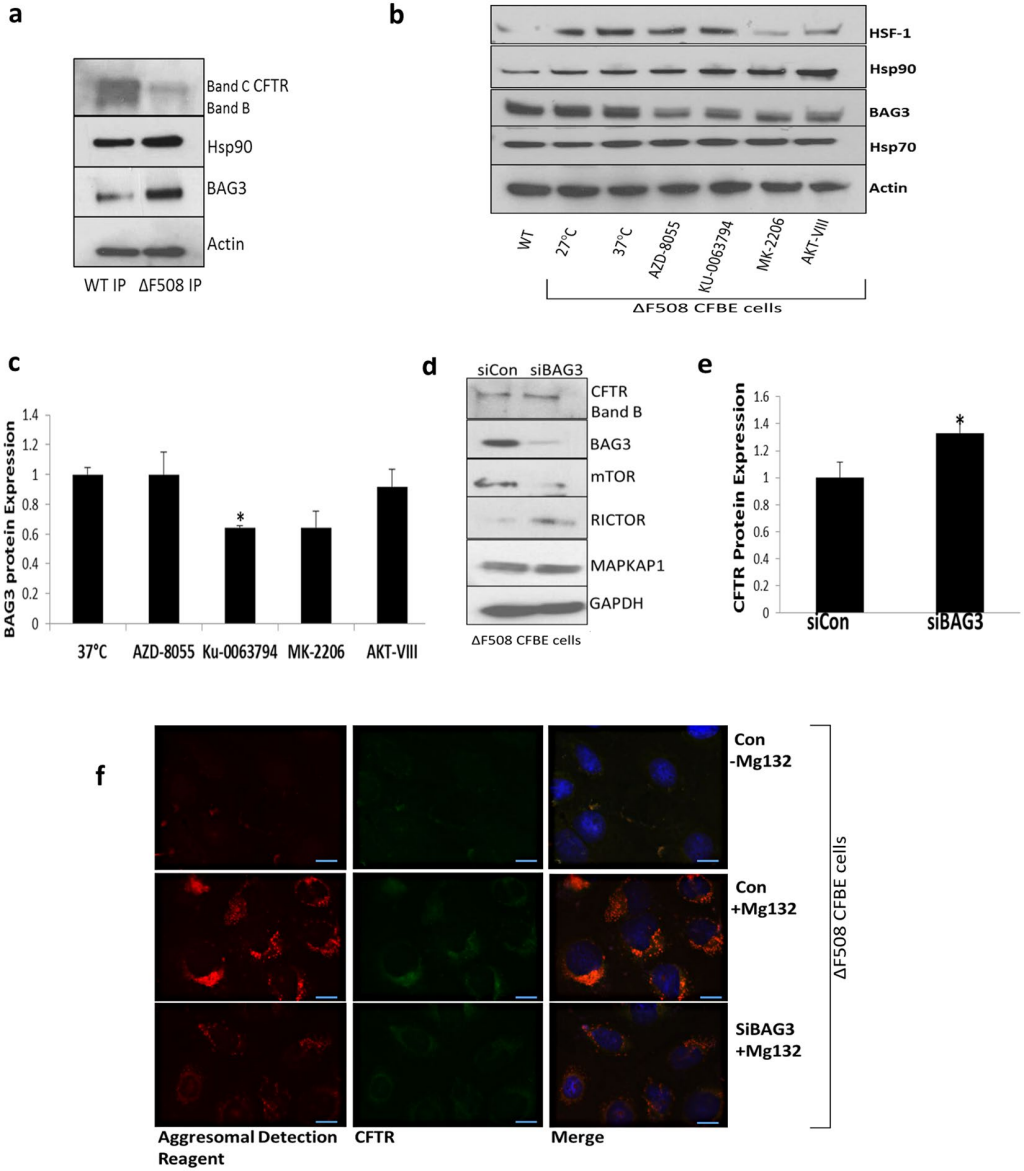
PI3K/AKT/mTOR inhibition increases ΔF508 CFTR stability through BAG3. (**a**) CFTR was immunoprecipitated from WT HBE41o- and ΔF508 CFBE41o- cells and immunoblotting was performed for Hsp90, BAG3 and β-actin**. (b**) ΔF508 CFBE41o- cells (37 °C) were treated with AZD-8055, KU-0063794, AKT-VIII and MK-2206 and the protein expression of Hsp70, Hsp90, BAG3 and HSF1 and actin was determined. WT HBE41o- cells and ΔF508 CFBE41o- cells (27 °C) acted as positive controls and ΔF508 CFBE41o- (37 °C) was a benchmark for comparison (**c)** A quantitative graph of BAG3 from ΔF508 CFBE41o- lysates, CFBE41o- cells (37 °C) were treated with AZD-8055, KU-0063794, AKT-VIII and MK-2206. The results shown are representative of three independent experiments; the histograms represent the average and the error bars represent the standard deviation of the means. Statistical significance was examined using a two tailed by paired T-test analysis. Asterisk *represents p ≤ 0.05 (**d**) Protein expression levels of CFTR, mTOR, RICTOR, MAPKAP1 and BAG3 were determined by immunoblotting in ΔF508 CFBE41o- cell lysates treated with control and BAG3 siRNA. (**e**) A quantitative graph of CFTR from ΔF508 CFBE41o- cell lysates treated with control (siCon) and BAG3 siRNA (siBAG3) was performed. Statistical significance was examined using a two-tailed paired T-test analysis. Asterisk* represents p < 0.05. (**f)** Fluorescent detection was used to visualise CFTR (green- 488 nm) and aggresomes (red- 549 nm) in ΔF508 CFBE41o- cells treated and untreated with proteasome inhibitor MG132 (10 µM) to validate the presence of aggresomes in these cells after MG132 treatment. Aggresome fromation in ΔF508 CFBE cells in the presence of siBAG3 relative to siControl under proteasomal inhibiton was then investigated. Nuclei were stained with DAPI (blue- 358 nm).

**Figure 6:**
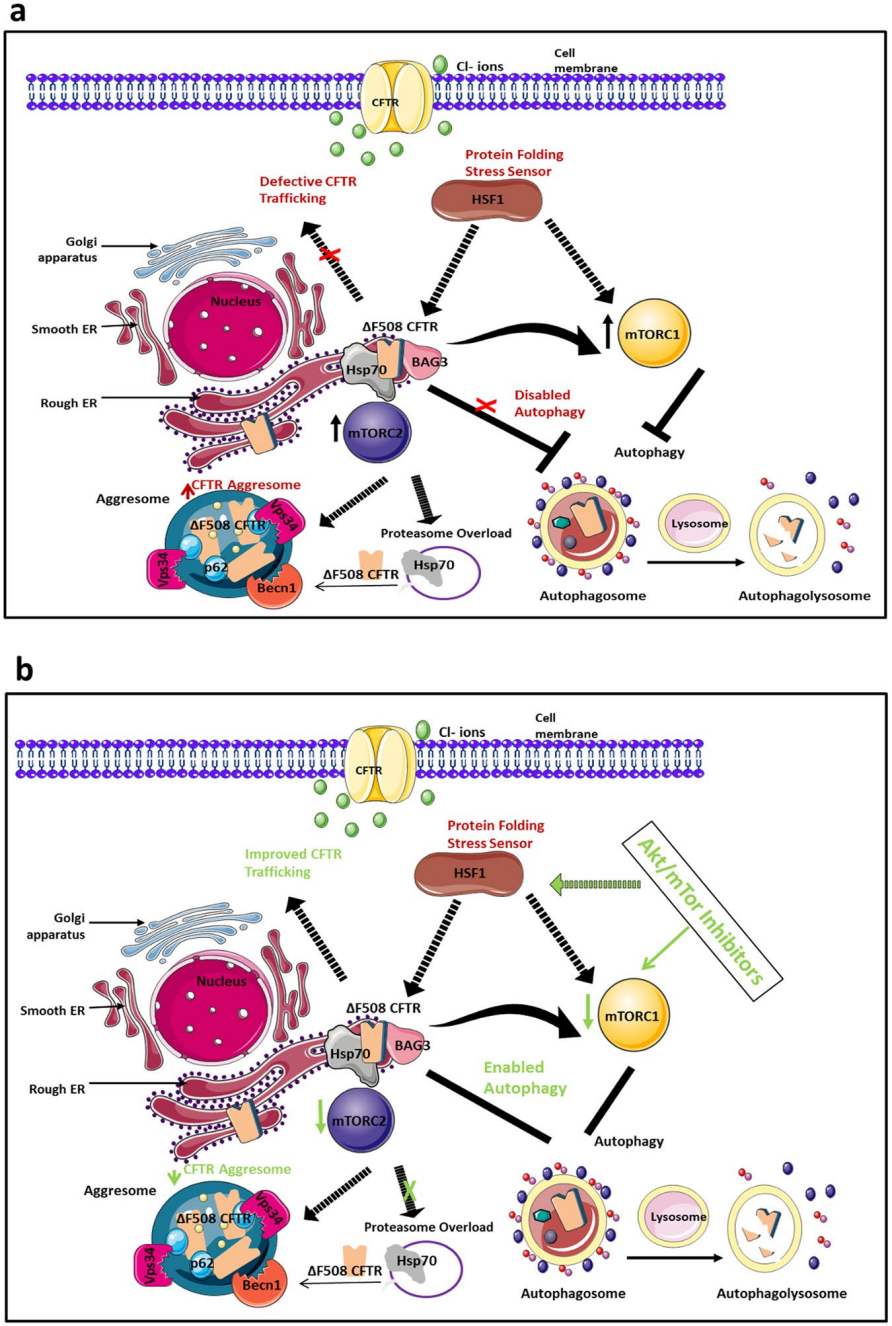
Regulation of ΔF508 CFTR stability by inhibitors to the PI3K/Akt/mTOR pathway. (**a**) The ΔF508 variant of CFTR is characterized by ER accumulation, misfolding and an inability to traffic correctly to the plasma membrane where it functions as a chloride channel. It is believed that ΔF508 CFTR is retained in the ER in a Hsp70/90-containing chaperone trap influenced by the stress response sensor Heat Shock Factor (HSF1). Degradation of CFTR by the ERAD machinery is favoured but when CFTR that cannot be degraded by the proteasome machinery, (proteasome overload), CFTR can be stocked in the cytoplasm in the form of aggresomes. Our results indicate that mTORC1/2 signalling is upregulated in ΔF508 CFBE cells which have a defective autophagy pathway. Defective autophagy has been connected to reduced aggresome clearance. We observed CFTR localisation within aggresomes in ΔF508 CFBE41o- cells under conditions of proteasome inhibition. (**b)** In the presence of inhibitors to the PI3K/AKT/mTOR (eg MK-2206) we observed a decrease in mTORC1/2 activation and a corresponding increase in autophagic activity. We detected an increase in CFTR stability/trafficking upon therapeutic intervention of this pathway. We also revealed that HSF1 and BAG3 expression (which is stimulated by HSF1) are potentially regulated by PI3K/AKT/mTOR inhibitors. These data suggest that selective PI3K/Akt/mTOR inhibitors, such as MK-2206, may improve CFTR stability by both restoring autophagy through inhibition of mTOR and through the HSF1/BAG3 axis which, in turn, can regulate autophagy and ΔF508 CFTR aggregates. This figure was produced, in part, by using Servier Medical Art, (www.servier.com/Powerpoint-image-bank).

CFTR misfolding is regulated by a core chaperone machinery^10^. We observed an increase in the association of specific chaperones with ΔF508 CFTR. Specifically, an increase in protein expression of BAG3 (Bcl2-associated Athanogene 3) and Hsp90 in ΔF508 CFTR immunoprecipitates was detected relative to the WT CFTR control (Fig. 5a). We hypothesized that inhibitors of the PI3K/Akt/mTOR pathway may additionally regulate CFTR expression by targeting the chaperone misfolding machinery. We examined the protein expression of HSP72, BAG3, Hsp90, and heat shock factor regulator (HSF1) in the presence of four inhibitors. Interestingly, we observed a decrease of the expression of BAG3 in the presence of the four inhibitors (Fig. 5b). After performing quantification in triplicate, we observed that MK-2206 (0.64 ± 0.15) and KU-0063794 (0.64 ± 0.16) exhibited the most substantial decrease in BAG3 expression relative to the 37 °C control (1 ± 0.06) (Fig. 5c). We next examined if BAG3 could regulate CFTR and mTOR expression. We visually observed modest differences in mTOR and CFTR protein expression upon BAG3 inhibition. (Fig. 5d). To confirm this observation, we quantified CFTR and observed a 1.3-fold significant increase in CFTR expression after gene silencing of BAG3 (p < 0.05) (Fig. 5e). As BAG3 has been reported to mediate aggresome-targeting of chaperoned substrates^28^ we hypothesized that inhibiting BAG3 may contribute to CFTR stability by reducing ΔF508 CFTR aggregates. We observed that CFTR localised to aggresomes in ΔF508 CFBE41o- cells treated with MG132 exhibited a modest reduction in ΔF508 CFTR upon inhibition with siBAG3 (Fig. 5f). A summary of our results before (Fig. 6a) and after inhibition of the PI3K/Akt/mTOR pathway (Fig. 6b) are illustrated in Fig. 6.

## Discussion

The biological pathways dictating the folding and function of ΔF508 CFTR provide a unique opportunity to identify new therapeutic targets in CF. In addition to the central core pathways regulating CFTR misfolding integrated biological networks exist that are sensitive to the status of the CFTR fold. To identify signalling pathways that potentially drive the CF disease phenotype and lead to novel therapeutic targets, we used a protein interaction profiling screen. This involved immunoprecipitation of the CFTR protein in bronchial epithelial cells expressing ΔF508 CFTR and WT CFTR coupled to mass spectrometry based proteomics. 324 and 322 proteins were identified to uniquely interact with ΔF508 and WT CFTR respectively. Using pathway analysis, we identified specific signalling pathways to be more significantly associated with the different phenotypes. A significant number of proteins involved in calveolar-mediated endocytosis were associated with WT CFTR and mediators of the ubiquitination pathways and mitochondrial dysfunction were associated with ΔF508 CFTR. These observations are consistent with previous studies where a ΔF508 CFTR specific interactome has been reported^10, 29^. Intriguingly, we observed an increase in the number of proteins associated with mTOR and downstream EIF4 signalling pathways.

mTOR, a highly conserved serine/threonine kinase, is a central regulator of cell growth and metabolism in eukaryotes^30^. mTOR is present in two functionally and structurally distinct multiprotein complexes and activates cell growth in response to nutrients, growth factors and cellular energy status^31^. mTOR is interconnected with the stress response pathways to maintain protein homeostasis and excessive activation of the protein is involved in age-related misfolding diseases^32^. Our data demonstrated an association of mTORC2 members, MAPKAP1 and RICTOR, with ΔF508 CFTR. Such associations have not been previously described, although mTORC2 complex members and members of the CFTR chaperone machinery (Hsp70) have been shown to form part of the mTORC2 complex under heat shock stress^33^. mTORC2 has also been shown to be localize to the ER where ΔF508 CFTR resides^34, 35^. Therefore, we hypothesised that association between ΔF508 CFTR and the mTORC2 complex may be mediated by chaperones and our protein interaction profiling of the RICTOR complex in WT HBE41o- and ΔF508 CFBE41o- cells showed clear interactions between RICTOR and the chaperone machinery, supporting this hypothesis (Supplementary Fig. S1). Our data support an overall induction in mTORC2 activity in ΔF508 CFBE41o- cells, as indicated by phosphorylation at serine 2481 which correlates with mTORC2 assembly^36^ and Akt-Ser473 phosphorylation, required for mTORC2 function^37^. Induction of mTORC1 activity was also observed in ΔF508 CFBE41o- cells, although differential activation of these individual pathways will require further investigation in CF.

Strategies aimed at manipulating peripheral signalling pathways that modulate proteostasis such as the mTOR pathway could represent a promising area of research in CF drug discovery. In order to determine if these pathways may be important for the correction of ΔF508 CFTR, we explored mTORC1/2 activity under established conditions of CFTR correction. Culturing cells at 26–30 °C promotes formation of fully glycosylated ∆F508 CFTR (band C), incorporation into the plasma membrane, and partial restoration of its channel activity^38^. Therefore, incubation of cells expressing ΔF508 CFTR at reduced temperature provides insights into temporal dynamics of interactions under partial correction. We observed a decrease of mTORC1/2 activity during temperature shift conditions, suggesting these pathways may be important for the correction of ΔF508 CFTR. We then hypothesised that inhibitors of PI3K/Akt/mTOR may promote stability of ΔF508 CFTR. Inhibitors of the PI3K/Akt/mTOR pathway are therapeutic targets in misfolding disease^39^ and the Akt inhibitor 10-DEBC has been previously shown improve ΔF508 CFTR stability in CF cells^40^. However, PI3K/Akt/mTOR signalling has not been explored in detail in ∆F508 CFTR expressing cells. We selected small molecules targeting the PI3K/Akt/mTOR pathway which included next generation inhibitors against mTORC1/2 and Akt (AZD-8055, MK-2206). Although fewer off-target effects have been shown with second generation inhibitors, such as MK-2206^41^, we used the lowest dosages that elicited a response to reduce off-target effects. Inhibition of the PI3K/Akt/mTOR pathway with inhibitors targeting different elements of the pathway demonstrated an increase in CFTR stability and expression.

We hypothesised that CFTR is potentially linked to mTOR signalling through autophagy. Although ER stress promotes autophagy to limit misfolded CFTR accumulation in the ER, autophagy has been shown to be defective in human CF airway epithelia. This is due to alterations in the autophagy pathway, which results in accumulation of misfolded CFTR in aggresomes^9^. In order to determine a mechanism of action in CF models, we investigated if the selected inhibitors of the PI3K/Akt/mTOR pathway could restore defective autophagy in CF cells. In mammalian cells, mTORC1 tightly regulates autophagy by suppressing phosphorylation-dependent inhibition of ULK1/2, which interacts with ATG13, an essential player in autophagosome formation^42^. Recent studies demonstrated Akt can directly regulate autophagy by phosphorylating beclin-1 in the VPS34 complex and that ΔF508 CFTR can be sequestered into aggresomes with beclin-1^43, 44^. We measured a panel of autophagosome formation and maturation markers in ΔF508 CFBE41o- cells and observed a strong induction of LC3B (II) and ATG family members with AKT-VIII and MK-2206. Our results also demonstrated an increase of CFTR co-localization with LC3 and a decrease in CFTR aggresomes with MK-2206, supporting previous studies that therapeutic restoration of autophagy reduces CFTR aggregation^9^ and improves ΔF508 CFTR maturation and trafficking. These results further support a role for mTOR signalling in maintaining protein homeostasis^32^.

With the intention of elucidating further mechanisms by which PI3K/Akt/mTOR may regulate CFTR stability, we investigated the role of chaperones associated with CFTR misfolding. Molecular chaperones play a role in regulating the autophagy pathway and influencing mTORC1 assembly in coordination with nutrient availability. Blockade of the PI3K/Akt/mTOR signalling axis was shown to attenuate the Heat Shock Factor (HSF1)-driven cellular heat shock stress response (HSR) in tumor cells^45^. Silencing of HSF1, the master regulator of the HSR in primary CF bronchial epithelial cells restores cellular protein folding and improves disease phenotype^11^. We hypothesized that PI3K/Akt/mTOR inhibitors regulate CFTR stability by influencing expression of Hsp70 and its co-chaperones that play essential roles in CFTR degradation^46^. Our results demonstrated an interaction between ΔF508 CFTR and the BAG proteins (1–3). BAG3 was of particular interest as it has been identified as a co-chaperone of Hsp70/Hsc70 that can target misfolded and aggregated proteins for selective autophagic degradation^47^. BAG3 has also been shown to be directly regulated by HSF1^48^. We found a decrease in the levels of BAG3 with inhibitors to the PI3K/Akt/mTOR pathway and hypothesised that BAG3 may stabilise ΔF508 CFTR by reducing the levels of ΔF508 CFTR aggresomes. In support of this, our results demonstrate a modest decrease in ΔF508 CFTR aggregates suggesting BAG3 may be a mechanistic target. These data suggest that selective PI3K/Akt/mTOR inhibitors, such as MK-2206, may improve CFTR stability by both restoring autophagy through inhibition of mTOR and through the HSF1/BAG3 axis which, in turn, can regulate autophagy and ΔF508 CFTR aggregates (Fig. 7).

In this study, we describe a novel role for mTORC1/2 regulation in ΔF508 expressing CF cells. We discovered that inhibition of the PI3K/Akt/mTOR pathway improved CFTR stability and suggest that this pathway merits further study as a therapeutic target in CF. We also showed that select inhibitors to the pathway exerted rescue effects by restoring autophagy and reducing ΔF508 CFTR aggregate formation. These findings further support a role for mTOR signalling in maintaining protein stability in misfolding disease.

## Electronic supplementary material

Supplementary Material

Supplementary Table 1

Supplementary Table 2
