# Supplementary material for: Targeting the PI3K/Akt/mTOR signalling pathway in Cystic Fibrosis
Source: Sci Rep. 2017 Aug 9;7:7642. doi: 10.1038/s41598-017-06588-z (PMC5550428; doi:10.1038/s41598-017-06588-z)

Targeting the PI3K/Akt/mTOR signalling pathway in Cystic Fibrosis

Reilly, R Mroz, MS Dempsey, E Wynne, K Keely, SJ McKone, EF Behl, C Hiebel, C Coppinger, JA

Supplementary Materials

All antibodies and inhibitors and their sources are listed in Supplementary Materials. All buffer compositions for CO-PIT, STAGE clean up, Immunoblotting, Immunofluorescence, LC-MS/MS and Short Circuit Analysis are also listed in table format (Tables 1.1-1.5)

Supplementary Table S1

Mass spectrometry analysis of the ΔF508 CFTR Interactome. The gene accession numbers, protein names, protein classes GO functions, score, and peptide numbers are displayed.

Supplementary Table S2

Mass spectrometry analysis of the WT CFTR Interactome. The gene accession numbers, protein names, protein classes GO functions, score, and peptide numbers are displayed.

Supplementary Table S3

Selected inhibitors to the PI3K/Akt/mTOR are listed along with their molecular target and references.

Supplementary Figure S1

RICTOR was immunoprecipitated from HBE41o- and CFBE41o- cells expressing WT and ΔF508 CFTR and the resulting RICTOR immunocomplexes were subjected to mass spectrometry analysis. The gene name, protein name and number of peptides identified of RICTOR and eight interactors are displayed in the table.

Supplementary Figure S2

ΔF508 CFBE41o- cells (37°C) were treated with increasing concentrations of AZD-8055, KU-0063794, AKT-VIII and MK-2206 and protein expression for CFTR was determined by immunoblotting.

Supplementary Figure S3

Subcellular localization of CFTR in ΔF508 CFBE41o- cells treated with MK-2206, KU-0063794 was detected by confocal microscopy.

Supplementary Figure S4

Co-localization of CFTR and LC3 in WT HBE41o- cells

Supplementary Figure S5

Co-localization of CFTR and aggresomes was investigated by confocal microscopy in ΔF508 CFBE cells treated with MK-2206.

Supplementary Materials

ABCAM: BAG3 antibody (ab86298)

Cell Signalling: mTOR (2983 P), mTOR ser2448 (5536 P), mTOR ser2481 (2974 P), total p70s6 kinase, p70s6 kinase thr389 (9234 P), total Akt, pAkt ser473 (9271 S), RICTOR (2114 P), RAPTOR (2280 P), Gβl (3274 P), Autophagy Antibody Sampler Kit #4445 containing: ATG3, ATG5, ATG12, Beclin-1, ATG16L and ATG7. HSP90 (4877 P), HSP70 (4872 P), Sin1 (12860 S) and anti-rabbit HRP (7074 P2).

Chemdea: AZD-8055 (CD0348)

GIBCO: Penicillin Streptomycin (15070), Foetal bovine serum (10270-106)

MERCK: Rapamycin *(*553210-100UG)

MERCK MILLIPORE: CFTR antibody M3A7 (05-583)

Nanotools: LC3 antibody (0231-100/LC3-5F10)

Santa Cruz: goat anti-mouse HRP (sc-2005), MAPKAP1 antibody (sc-393166)

Selleck Chemicals: MK-2206 (S1078), VX-770 (S1144), VX-809 (S1565)

Sigma Aldrich: Ly-294002 (L9908), AKT-VIII (A6730), genistein (G6649), CFTR (inh) 172 (C2992), Forskolin (F3917)

Thermo fisher scientific: Minimum essential medium with L-glutamine (31095-029), goat anti-mouse Alexa Fluor® 594 (A-11005), anti-rabbit Alexa Fluor® 488 (A 11034)

TORCIS: KU-0063794 (3725), PP 242 (4257), 10-DEBC (2558)

UNC Chapel Hill: CFTR 660 antibody, CFTR 596 antibody

Buffer Composition

Co-PIT

**Solutions**	**Reagent Composition**
Dynabeads	200 ul per IP
Crosslinking buffer	0.2 M (1.52 ml) triethanolamine in 50 ml PBS pH 8.2
Crosslinking buffer: Dimethyl pimelimidate (DMP)	0.2 M (1.52 ml) triethanolamine, 0.025 M DMP in 50 ml PBS pH 8.2
Quenching/blocking buffer	0.2 M (610 µl) ethanolamine in 50 ml PBS, pH 8.2
Antibody Elution buffer	1 M (3.75 g) glycine in 50 ml H_2O pH 2.5
Lysis buffer	1% (500 µl) Surfact-Amps® NP-40, protease and phosphatase inhibitor mix in 50 ml PBS
Protein complex elution buffer	8 M (4.8 g) urea, 0.5 % (100 µl) Surfact-Amps® NP-40 in 10 ml PBS

Table 1.1: Solutions used in Immunoprecipitation.

Stop and Go protocol; protein digest clean-up (STAGE)

**Solutions**	**Reagent composition**
Buffer A	0.1 % 20 µl trifluoroacetic acid (TFA) in 19.9 ml ddH_2O
Buffer B	50 % 10 ml acetonitrile, 0.1 % 20 µl trifluoroacetic acid, 50 % 9.9 ml ddH_2O
Buffer C	1 % 200 µl trifluoroacetic acid in 19.98 ml ddH_2O

Table 1.2 Solutions used in STAGE protocol.

LC-MS/MS Buffers

**Solutions**	**Reagent composition**
Buffer A	Buffer A: 97 % water, 2.5 % acetonitrile, 0.5 % acetic acid
Buffer B	Buffer B: 97 % acetonitrile, 2.5 % water, 0.5 % acetic acid.

Immunoblotting

**Solutions**	**Reagent composition**
Lysis buffer	1 % SDS protease and phosphatase inhibitor mix
Running buffer	0.025 M (3.03 g) Tris base, 0.192 M (14.04 g) glycine, 0.1 % (1 g) SDS, pH 8.5 in 1 L H_2O
Transfer buffer	0.025 M (6.06 g) Tris base, 0.192 M (28.8 g) glycine, 30 % methanol in 2 L H_2O
10X Tris-buffered saline (TBS)	0.171 M (12.1 g) Tris, 1.44 M (42 g) NaCl in 500 ml H_2O
1x Tris-buffered saline tween®20 (TBS-T)	100 ml of 10 X TBS, 0.1 % Tween®20 in 900 ml H_2O
5x SDS sample buffer	50 mM Tris, 4 % w/v SDS, 20 % glycerol, 10 % v/v β-mercaptoethanol, 0.02 % w/v bromophenol blue
Protein stain	0.1 % w/v ponceau s stain in 10 % acetic acid

Table 1.3: Solutions used in immunoblotting protocol.

Immunofluorescence

**Solutions**	**Reagent composition**
Fix solution	0.45 g of PFA, 200 µl 1 M NaOH, 15 ml PBS
Blocking buffer	2 g BSA, 100 µl triton X-100, 50 ml PBS
Antibody dilution solution	0.5 g BSA, 100 µl triton X-100, 15 ml PBS

Table 1.4: Solutions used in immunofluorescence protocol.

Short circuit current analysis

**Reagent**	**Concentration (mM)**	**Location in chamber**
NaCl	120	Basolateral
$NaHCO_3$	25	Bilateral
KH_2PO_4	3.3	Bilateral
K_2HPO_4	0.8	Bilateral
$MgCl_2$	1.2	Bilateral
$CaCl_2$	1.2	Bilateral
Glucose	10	Bilateral
Na gluconate	120	Apical

Supplementary Table1 & 2- Attached as Excel Files

Supplementary Table 3

Compound	Primary Target	Source	Reference
Rapamycin	Binds to FKBP12 and complex prevents TOR phosphorylation	Calbiochem, Merck	Heitman *et al* (1991) Targets for cell cycle arrest by the immunosuppressant rapamycin in yeast. *Science* 23 Aug 1991: Vol. 253, Issue 5022, pp. 905-909
Ku-0063794	Inhibits mTORC1 and mTORC2, inhibits activation of Akt, S6K and SGK.	Tocris	Garcia-Martinez *et al* (2009) Ku-0063794 is a specific inhibitor of the mammalian target of rapamycin (mTOR). Biochem.J. *421* 29.
PP242	ATP competitive mTORC1 and mTORC2 inhibitor.	Tocris	Feldman *et al* (2009) Active-Site Inhibitors of mTOR Target Rapamycin-Resistant Outputs of mTORC1 and mTORC2. PLoS Biol.2009 Feb 10;7(2):e38.
AZD8055	ATP competitive mTORC1 and mTORC2 inhibitor.	Chemdea	Chresta *et al* (2010) AZD8055 Is a Potent, Selective, and Orally Bioavailable ATP-Competitive Mammalian Target of Rapamycin Kinase Inhibitor with In vitro and In vivo Antitumor Activity. Cancer Res. 2010 Jan 1;70(1):288-98.
10-DEBC	Akt with downstream effects on mTOR and S6K.	Tocris	Thimmaiah *et al* (2005) Identification of N^{10}-substituted phenoxazines as potent and specific inhibitors of Akt signaling. J.Biol.Chem. (2005) *36* 31924
Ly-294002	Phosphoinositide 3-kinase inhibitor. Targets mTOR via ATP binding site.	Sigma Aldrich	Chris J. Vlahos *et al* (1994) A specific inhibitor of phosphatidylinositol 3-kinase, 2-(4-morpholinyl)-8-phenyl-4H-1-benzopyran-4-one(LY294002). Biol Chem. 1994 Feb 18;269(7):5241-8.
MK2206	Allosteric Akt inhibitor by stopping autophosphorylation of Akt at T308 and Ser473.	Selleck Chemicals	Hirai *et al* (2010) MK-2206, an Allosteric Akt Inhibitor, Enhances Antitumor Efficacy by Standard Chemotherapeutic Agents or Molecular Targeted Drugs *In vitro* and *In vivo.* Mol Cancer Ther July 2010 9; 1965
AKTVIII, AKT1/2	Allosteric Akt inhibitor by binding PH domain.	Sigma Aldrich	Wu *et al* (2010) Crystal Structure of Human AKT1 with an Allosteric Inhibitor Reveals a New Mode of Kinase Inhibition. PLoS One. 2010; 5(9): e12913.

Supplementary Figure S1

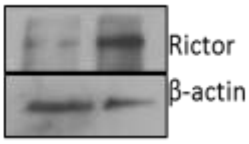

GENE NAME	Protein Name	WT Rictor IP Peptide No.	ΔF508 Rictor Peptide No.
RICTR-HUMAN	Rapamycin Insenstive companion of mTOR	15	25
MTOR_HUMAN	Serine/threonine-protein kinase mTOR	1	1
SIN 1_HUMAN	Target of rapamycin complex 2 subunit MAPKAP	2	6
CFTR_HUMAN	Cystic fibrosis transmembrane conductance regulator	0	0
HS90B_HUMAN	Heat Shock Protein Hsp90beta	5	6
HSP7C_HUMAN	Heat Shock Cognate 71	14	17
HSPB1_HUMAN	Heat Shock Beta-1	1	8
BAG3_HUMAN	BAG family molecular chaperone regulator 3	0	1
ACTG_HUMAN	Actin Cytoplasmic 2	30	42

Supplementary Figure S2

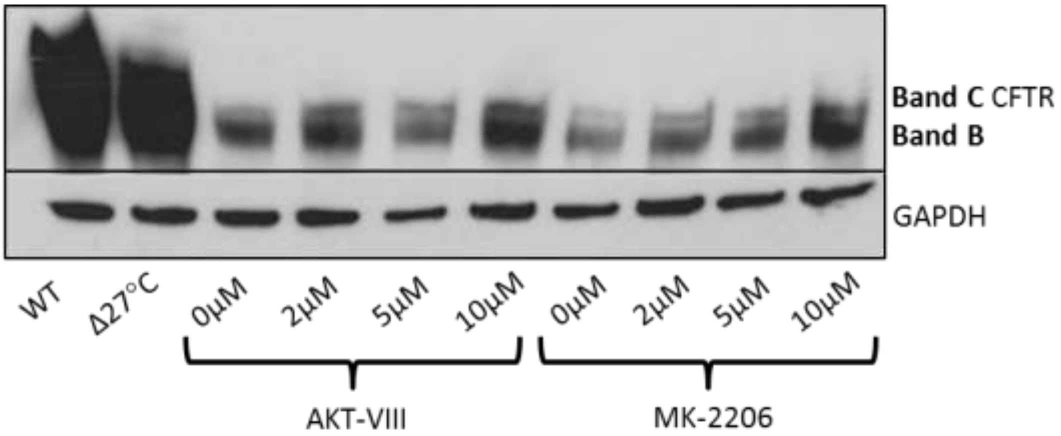

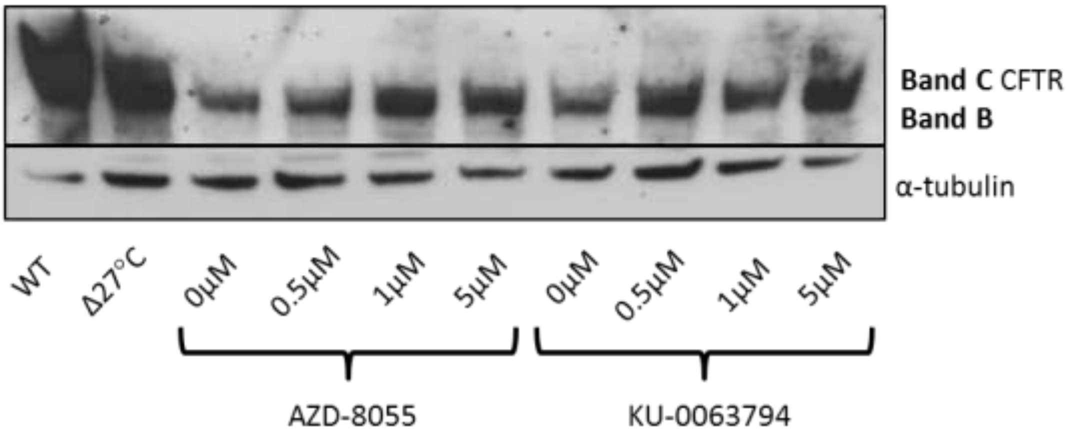

Supplementary Figure S3

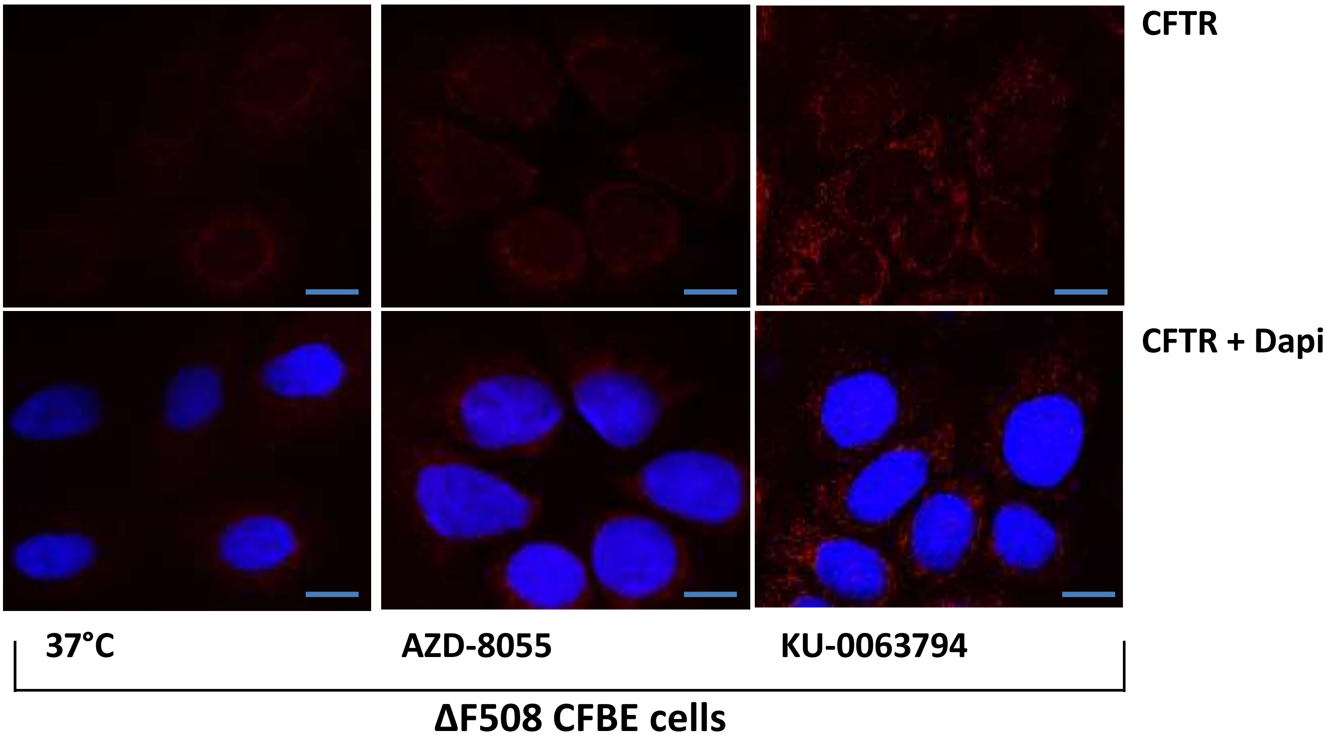

Supplementary Figure S4

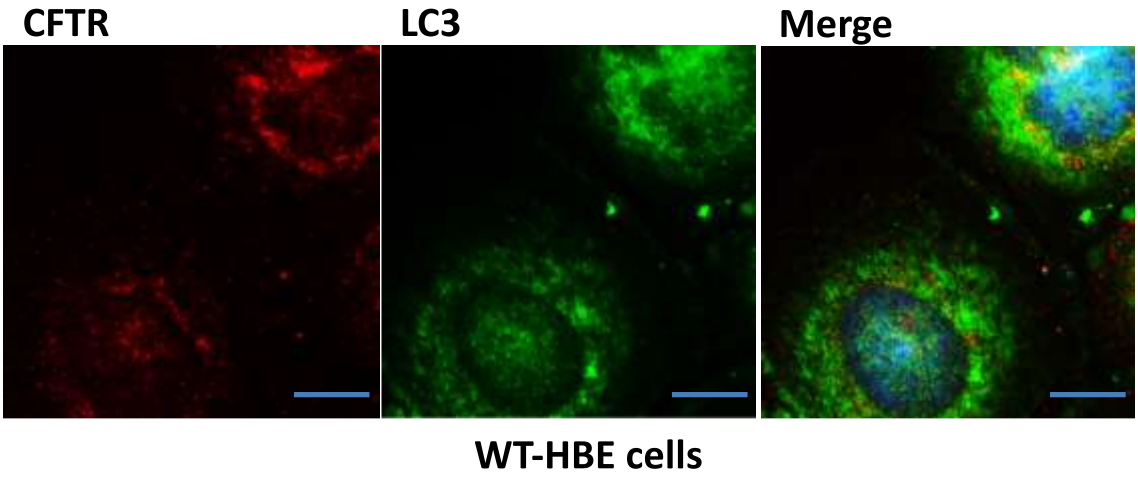

Supplementary Figure S5

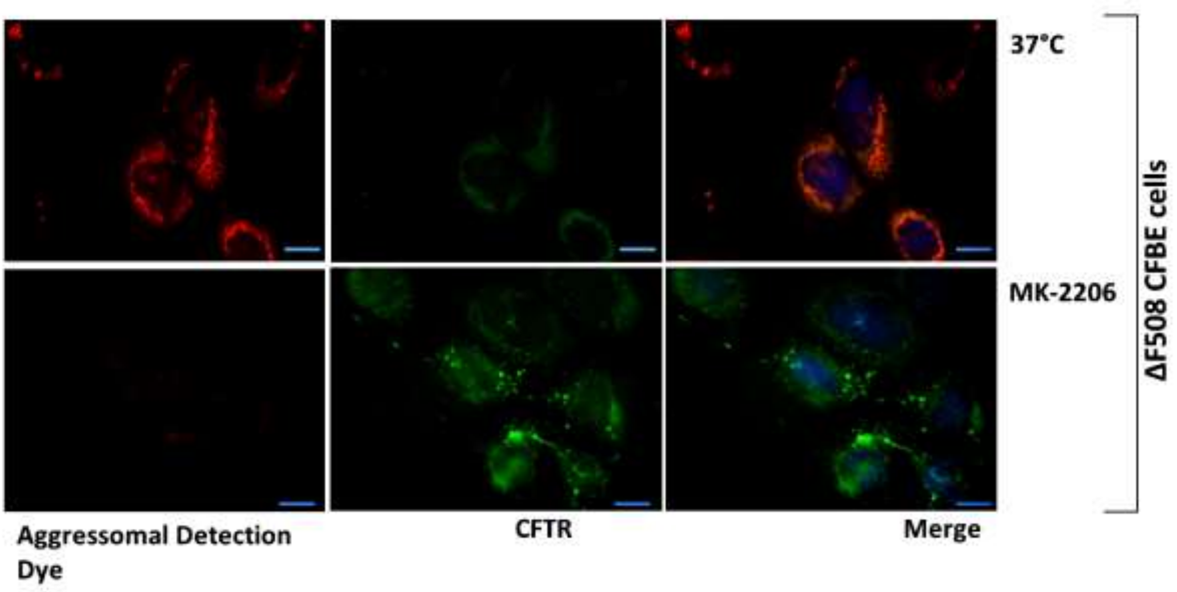

Supplement: Supplementary file 1 — Supplementary Material [file 41598_2017_6588_MOESM1_ESM.pdf]
